# Effects of Goldblatt hypertension on rats’ hippocampal cholinergic system

**DOI:** 10.1515/tnsci-2022-0215

**Published:** 2022-04-20

**Authors:** Hamid Sepehri, Farzaneh Ganji, Zahra Nazari, Marzieh Vahid

**Affiliations:** Department of Physiology, Neuroscience Research Center, Golestan University of Medical Sciences, Gorgan, Iran; Department of Biology, Faculty of Science, Golestan University, Gorgan, Iran

**Keywords:** Goldblatt hypertension, AT receptors, hippocampus, ChAT

## Abstract

**Background:**

The classical renin-angiotensin system (RAS) has an important role in the cardiovascular system and water homeostasis in the body. Recently, the existence of RAS with all of its components has been shown in the mammalian brain. RAS participates in many brain activities, including memory acquisition and consolidation. Since the cholinergic neurotransmission in the hippocampus is crucial for these functions, this study aims to evaluate the hippocampal angiotensin receptors (ATs) and choline acetyltransferase (ChAT) mRNA in the renovascular hypertensive rats in captopril- and losartan-treated hypertensive rats.

**Methods:**

The rats were randomly divided into four groups of eight animals; sham, Goldblatt two kidney one clip (2K1C) hypertensive rats and Goldblatt 2K1C hypertensive rats received 5 mg/kg captopril and Goldblatt 2K1C hypertensive rats received 10 mg/kg losartan. After 8 days of treatment, the rats were sacrificed and angiotensin-converting enzyme (ACE), ChAT, AT1, and AT2 receptor mRNAs in the hippocampus of rats were assessed by real-time PCR. The Morris water maze test was applied to measure the cognitive functioning of the rats.

**Results:**

Hypertensive rats showed impaired acquisition and memory function in the Morris water maze test. Treatment with ACE inhibitor (captopril) and AT1 receptor antagonist (losartan) reversed the observed acquisition and memory deficit in hypertensive rats. Overexpression of AChE, AT1, and AT2 and low expression of ChAT were noted in the hippocampus of rats with Goldblatt hypertension compared with that of the sham group. Treatment with captopril significantly reversed these changes, while treatment with losartan slightly reduced the mentioned effects.

**Conclusion:**

The memory-enhancing effect of captopril in renovascular hypertensive rats might lead to increased hippocampal ChAT expression.

## 1 Introduction

The correlation between hypertension, cognitive impairment, and neural modifications has been well demonstrated [1,2,3]. The renin-angiotensin system (RAS) might help the induction of memory impairment in hypertension [4]. Several studies have shown the role of brain RAS in the cognitive function [5,6,7,8]. Clinical works have revealed that angiotensin-converting enzyme (ACE) inhibitors lower blood pressure and improve cognitive function in hypertensive individuals [9,10]. Besides, high levels of angiotensin II (Ang II) have been identified in the hippocampus that plays a role in the acquisition and recall of memories [11,12]. The angiotensin type 1 (AT1) and angiotensin type 2 (AT2) receptor mRNA expression in mature rat hippocampus have also been reported [13]. Srinivasan et al. reported a correlation between AT1 receptor blockers and a considerable decrease in the occurrence and the development of dementia and Alzheimer’s diseases [14]. However, the neurochemical systems underlying the cognitive effects of RAS in the hippocampus are not fully understood. Acetylcholine is a significant neurotransmitter that is involved in cognition and memory. Several works have revealed a relationship between memory deficit and the weakening of cholinergic brain function [15,16,17]. Central cholinergic deficit is intensely related to some neurodegenerative diseases, such as Parkinson’s or Alzheimer’s diseases [18,19]. Some studies also indicated an interaction between RAS and the brain cholinergic system [20,21]. These studies suggest that spatial memory formation, the cholinergic system, and cerebral blood flow are affected by Ang II via the AT1 receptor [1,2,3,4,5]. Surprisingly, it has been observed that the hippocampus contains cholinergic neurons [6]. It has been shown that cerebral hypoperfusion leads to memory impairment, alters cholinergic indices including choline acetyltransferase (ChAT), and decreases the levels of hippocampal acetylcholine, which can be alleviated by ACE inhibitors [7]. ChAT along with acetylcholine esterase (AChE) are markers for the functional state of cholinergic neurons in the brain [8]. In this study, we evaluated the effects of RAS on the hippocampal cholinergic system in two-kidney, one-clip (2K1C), Goldblatt hypertensive rats.

## 2 Materials and methods

### 2.1 Animals and treatments

**Figure 1:**
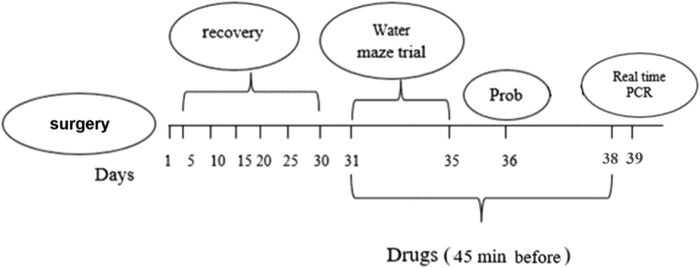
Diagram of experimental protocol for drug administration, surgery, behavioral, and gene expression analysis.

Three-month-old Wistar rats weighing 250–300 g were used in the study. The animals were fed ad libitum and were maintained at a controlled temperature (23 ± 1°C) and under a 12 h light/dark cycle. The ethical committee of the Golestan University of Medical Sciences approved all protocols for animal experimentation (irgoums.rec.1395.42). Animals were accidentally distributed into four groups of eight: the sham-operated control group (nonhypertensive rats), hypertensive group (Goldblatt 2K1C renal hypertensive rats [RHR]), Goldblatt 2K1C RHR receiving captopril (RHR + captopril), and Goldblatt 2K1C RHR receiving losartan (RHR + losartan). Normal saline was given to the sham group as a vehicle. Losartan at a dose of 5 mg/kg and captopril at a dose of 10 mg/kg were administered 45 min before testing for 8 consecutive days [9,10]. The drugs were dissolved in distilled water and used intraperitoneally at a certain volume of 1 mL/kg of body weight. Figure 1 shows a diagrammatic representation of the experimental protocol for drug administration, surgery, behavioral studies, and gene expression analysis.

**Figure 2:**
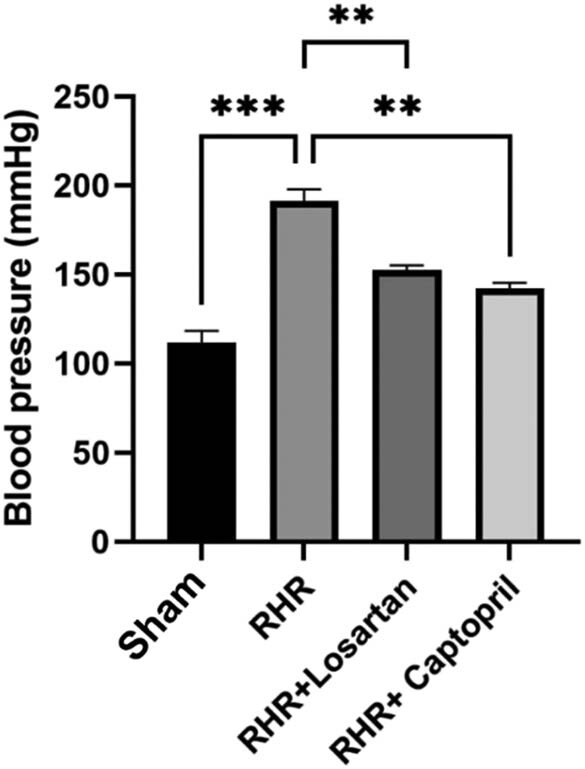
Systolic blood pressure levels of groups. Each group consists of eight animals. Treatments were started 3 weeks after surgery and continued for 8 consecutive days. Blood pressure levels were measured at the end of the study. RHR: renal hypertensive rats. Data are expressed as mean ± SEM. Data are analyzed with ANOVA followed by Tukey multiple comparison tests. RHR rats showed a significant higher systolic blood pressure compared to the control group. Both of the treatments significantly decreased the systolic blood pressure levels (***P < 0.001; **P < 0.01).


Hypertension was surgically induced in nonfasting rats using the renal occlusion method as described by Goldblatt et al. [11]. Intraperitoneal injection of chloral hydrate (400 mg/kg) was used to induce surgical anesthesia. Lumbar decompression surgery was performed under sterile conditions, in which the left kidney was exposed, and the anterior branch renal artery was occluded with aneurysm clips. The skin and muscle were sutured, and the rats were housed alone until recovered from the surgical wounds. Postoperative treatment included daily administration of amoxicillin (10 mg/kg) and ibuprofen (100 mg/kg) for 5 days. Then, the animals were housed in colony cages for acclimatization. The rats in the sham group underwent the same surgical procedure except for clamping of the renal artery. A tail-cuff blood pressure recorder (IITC Life Science, USA) was used to determine the systolic blood pressure before behavioral testing. Figure 2 shows the mean systolic pressure of the groups.

**Ethical approval:** The research related to animals’ use has been complied with all the relevant national regulations and institutional policies for the care and use of animals. The ethical committee of the Golestan University of Medical Sciences approved all protocols for animal experimentation (IRGOUMS.REC.1395.42).

### 2.2 Morris water maze experiment

The Morris water maze (MWM) used a container of a black circular pool (160 cm diameter and 60 cm high) filled with water (30 cm depth) with a temperature of 24 ± 2°C. The pool was separated into four quadrants randomly labeled northeast, northwest, southeast, and southwest. A submerged plexiglass platform (10 cm × 10 cm) was hidden 1 cm below the water surface and fixed in the center of the northwest quadrant. The animals received four sessions of training with the hidden platform for 5 days, with 60 s intersession intervals. Each session started by putting the rat at one of the three start points while facing the wall of the pool. The start location was changed in each training session. The training session was finished when the animal entered the platform. The rat was replaced on the platform for 15 s if it was unable to find the platform within the 60 s. In the acquisition of the spatial navigation task, the groups performed one session of four trials each day (day 1–5; trial 1–20). Spatial memory was assessed in a probe trial on the sixth day (trial 21). The platform was removed, and rats were permitted to swim for the 60 s. A computerized video tracking system (Maze router, Urmia Instruments Inc) was used to monitor the path of the animals in the maze. Parameters including the latency (the time it takes to reach the platform), swimming path length (SPL), and swimming speed in the training trials were measured. During the trial probe, the percentage run time in the quadrant of the water bath where the concealed platform had been located in the training trials was measured.

### 2.3 Quantitative RT-PCR

Quantitative RT-PCR (Q-PCR) was done to assess the result of treatment with captopril and losartan on ACE, ChAT, and AT1 and AT2 receptors expression in the hippocampal tissues of hypertensive rats. After the accomplishment of all behavioral tests, the rats were sacrificed by decapitation, and the brain hippocampal tissues were extracted. Total RNA was extracted from the hippocampal tissues using an RNA extraction kit (Gena Bioscience) and then stored at −70°C for cDNA synthesis. NanoDrop ND-1000 spectrophotometer was used to measure the RNA concentration and the purity of the samples. cDNA synthesis was conducted in a 20 µL solution containing 1 μg of DNaseI-treated total RNA and by the PrimeScript RT Reagent kit based on the manufacturer’s instructions. Gene-specific primers were designed using the PerlPrimer software (Bio-Rad, USA), and the oligonucleotide sequences are listed in Table 1. All PCR experiments were performed in duplicate for each cDNA sample using the Applied Biosystems 7300 Real-Time PCR System (Life Technologies) and the SYBR-Green PCR Master Mix kit (TaKaRa), according to the manufacturer’s protocol. B-Actin was used as the housekeeping gene, and the hippocampus cDNA of control rats was used as a calibrator. Relative gene expression was calculated using the 2-∆∆Ct method. All Q-PCR experiments were done in triplicate.

**Table 1:** Sequence of the primers used in the RT-PCR experiment

Genes	Forward primer	Reverse primer	PCR product size	GenBank accession no.
β-Actin	AAGATCAAGATCATTGCTCCTC	CTCAGTAACAGTCCGCCT	169	NM_031144.3
ACE	TTGACGTGAGCAACTTCC	CAGATCAGGCTCCAGTG	191	NM_012544.1
ChAT	TCATTAATTTCCGCCGTCTC	AGTCCCGGTTGGTGGAGTC	177	XM_017600025.1
AT1	CAGCTTGGTGGTGATTGTC	GCCATCGGTATTCCATAGC	146	XM_008760879.2
AT2	TAGTCTCTCTCTTGCCTTG	CTGACCTTCTTGGATGCTC	217	XM_006257432.3

### 2.4 Data analysis

The statistical analyses were done by GraphPad Prism 9 and SPSS v. 18.0, and the data were presented as mean ± standard error of the mean. Statistical comparisons between groups were performed depending on the normality of distribution and variances equivalence between groups. In cases of equal variance and normal distribution, the data were analyzed by one- or two-way analysis of variance (ANOVA), including repeated-measures ANOVA, according to the experimental design. For comparisons between groups and within days, one-way ANOVA was used, followed by Tukey’s honestly significant difference test for the *post hoc* analysis. If groups of data failed tests for normality and equal variance, results were conducted by the nonparametric test. The results were considered significant when *P* < 0.05.

## 3 Results

### 3.1 Blood pressure

After the treatments were ended, the tail-cuff method was used to measure the systolic blood pressure indirectly. The rats of each group were placed in a heated chamber (35°C) for 10 min, and then, they were positioned in individual plastic restrainers. Tails of the rats were wrapped with cuff (IITC Life Science, USA). To prevent stress-induced changes in the blood pressure levels, 1 week before the first blood pressure recording, the rats were trained daily by blood pressure measurement. The blood pressure levels were recorded at least 10 times, and the average of 5 of them was used (Figure 2).

### 3.2 Water maze learning

**Figure 3:**
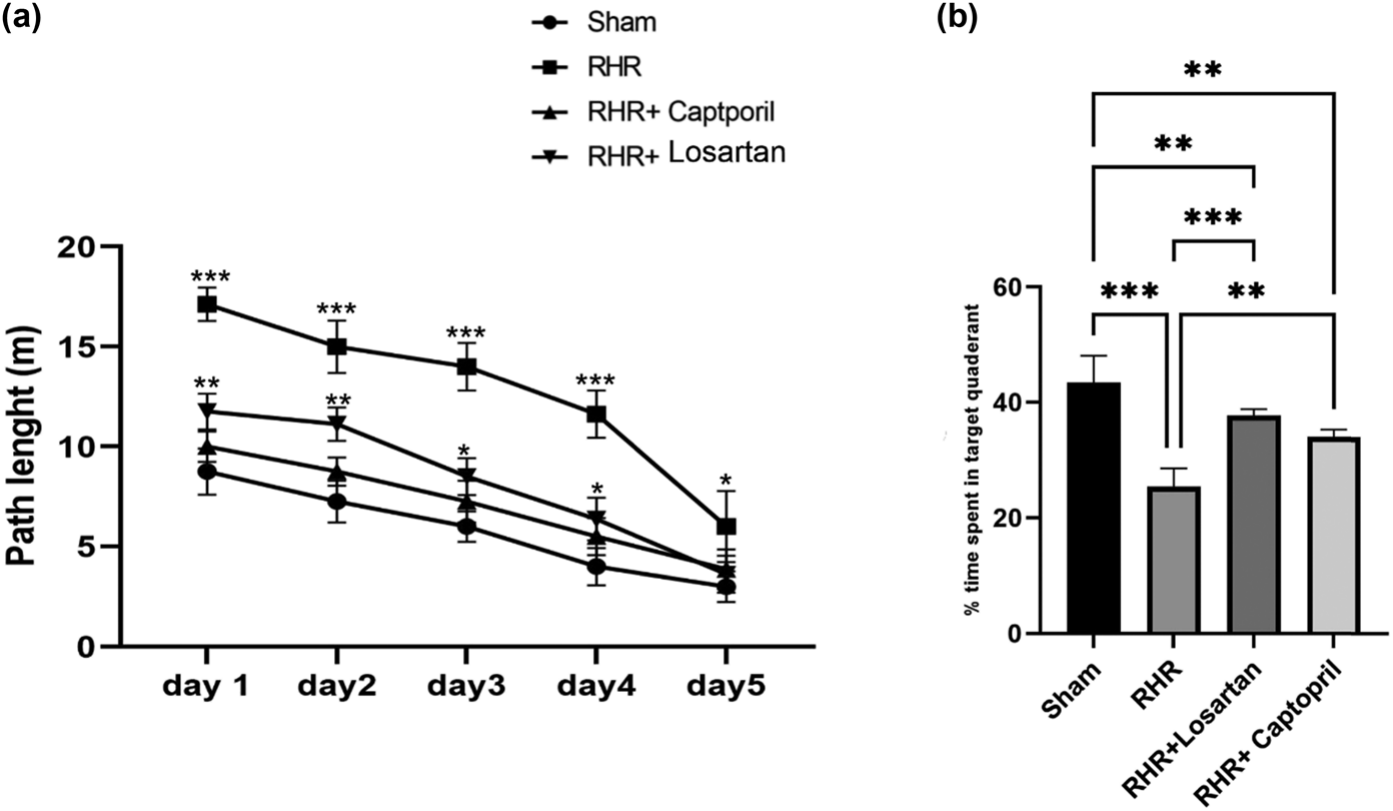
(a) Escape latency to reach the platform in each day during five training days or acquisition trials. The two-way ANOVA repeated-measure test followed by Tukey is used to analyze the data. (b) Time spent in target quadrant during the probe trial on day 6. In the probe-trial test, one-way ANOVA revealed that RHR rats spent a short time in the target quadrant compared to the sham group (*P* < 0.001). (Tukey pairwise multiple comparison test comparing percent search time in target quadrant versus each of the other quadrants after one-way ANOVA.) Losartan- and captopril-treated hypertensive groups spent more time in the target quadrant compared to the RHR group (*P* < 0.001). RHR, renal hypertensive rats; Sham, sham operated control; Asterisks indicate a significant difference from the sham (***P < 0.001, **P < 0.01, and *P < 0.05). Data are expressed as mean ± SEM (*n* = 8).

To assess hippocampus-dependent learning and memory, we measured the rats’ latency to find the platform over 5 training days and time they spent in the target quadrant during a probe trial on the sixth day in the MWM task. Figure 3 represents the MVM test results. The ability to find the platform was improved significantly in all animals over the 5 days of acquisition (*P* < 0.05). A two-way repeated-measures ANOVA analysis indicated a significant decrease in escape latency to find a hidden platform in all experimental groups during acquisition trials; *F*(3,140) = 186.12, *P* < 0.001 (Figure 3a). The subjects treated with captopril and losartan had improved learning and traveled a shorter distance to find the escape platform when compared with the RHR group (*P* < 0.05; Figure 3a). The probe trial data on day 6 presented that the average time spent in the target quadrant, where the hidden platform was formerly placed, meaningfully increased in subjects treated with captopril and losartan compared to the sham and control rats (Figure 3b).

### 3.3 Analysis of mRNA expression

#### 3.3.1 Analysis of mRNA expression

**Figure 4:**
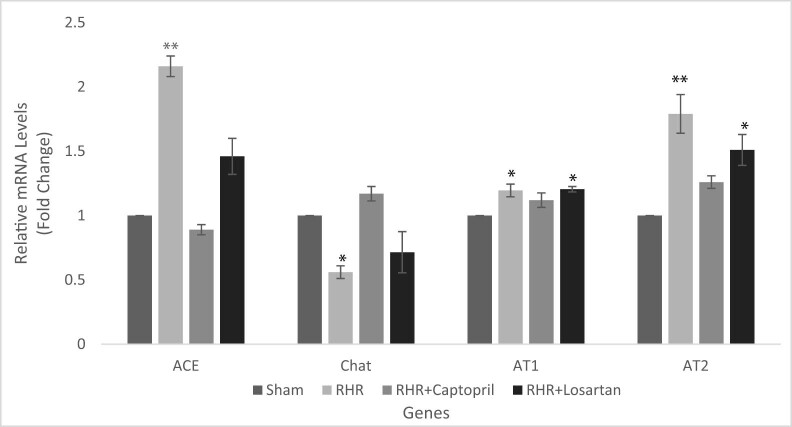
Q-PCR analysis of ACE, ChAT, AT1, and AT2 expression in the hippocampus of hypertensive and control rats; mRNA levels were measured using gene-specific primers, and the values were normalized to β-actin expression (*P < 0.05, **P < 0.01, mean ± SD, *n* = 5).

We assessed whether the expression levels of ACE, ChAT, AT1, and AT2 in hippocampus tissue were affected by hypertension in adult rats (Figure 4). Based on the results of the Q-PCR experiment, the expression of ACE was upregulated in the hypertensive rats compared to the controls (*P* < 0.05). We observed a more than twofold increase in ACE expression in the hypertensive group. This elevated level of ACE mRNA was significantly reversed after captopril treatment (fold change: 0.89). Although treatment with losartan also reduced these changes (*P* < 0.05), ACE was still significantly overexpressed compared with the controls (fold change: 1.46).

Our group also evaluated the ChAT mRNA expression following hypertension and treatment with captopril and losartan. The results showed about 46% decrease in the ChAT level in the hypertensive rats compared with the controls (*P* < 0.05, fold change: 0.56). While captopril treatment improved the ChAT levels in the hippocampal tissues of hypertensive rats (fold change: 1.17), losartan did not significantly affect this gene expression, indicating that captopril is more effective for modulating the ChAT levels.

Furthermore, the AT1 receptor was significantly overexpressed in hypertensive rats compared to control rats (*P* < 0.05, fold change: 1.19). Captopril treatment significantly lowered the expression of AT1 in the hypertensive rats, while the expression levels of this gene were not significantly different between the surgery and losartan groups.

Conversely, the analysis of the AT2 receptor mRNA showed significant overexpression in the hippocampus of hypertensive rats compared with the controls (*P* < 0.01, fold change: 1.79). Although the treatment with losartan decreased the level of AT2 expression (fold change: 1.51), the maximum decrease was noted after treatment with captopril (fold change: 1.26). Figure 4 demonstrates the relative ACE, ChAT, AT1, and AT2 receptors mRNA expression in the hippocampus of rats in all study groups.

## 4 Discussion

In the present study, we evaluated the effects of hypertension on the spatial memory and hippocampal cholinergic activity in a rat model of renovascular hypertension. The results of the MWM experiment indicated that hypertensive rats had impaired acquisition and memory, which was reversed after the treatment with captopril and losartan. There is some evidence to support an interaction between angiotensin and the cholinergic system in the rat brain [1,12,13,14]. Our findings are in agreement with the results of an in vitro study by Srinivasan et al. Pereira et al. also suggested that the ACE2/angiotensin axis in the brain is crucial for cognitive function [15]. Hence, blockade of angiotensin may have beneficial cognitive effects in the hypertensive state. The mechanisms of the nootropic effects of ACE inhibitors and angiotensin receptor blockers are not fully understood. Raghavendra et al. attributed the memory-enhancing effect of captopril and losartan to their effect on the cholinergic system [3]. In addition, Kułakowska et al. have suggested an antidopaminergic role for losartan and have suggested that losartan’s effects in enhancing conditioned memory may be related to its inhibitory role in dopamine release [16]. One more possibility is the action of these drugs to prevent oxidative stress [17].

Our results of RT-PCR indicated that ACE is overexpressed in the hippocampus of rats with renal hypertension. Similar to our findings, Cangussu et al. noted the increased ACE and AT1 receptor mRNA expression in the hypothalamus of Goldblatt hypertensive rats [18]. Although the circulating Ang-II peptides are too large to cross the blood–brain barrier (BBB) and act in the hippocampus, the hypertension-induced BBB disruption allows the circulating Ang-II to gain access to the neural brain regions [19]. Captopril treatment reversed the ACE mRNA expression almost to the normal levels. We observed that Goldblatt hypertension upregulated the expression of AT1 and AT2 receptors in the hippocampus of rats. Both AT1 and AT2 receptors are expressed at low levels under physiological conditions. AT1 receptor is involved in cerebral circulation and the regulation of inflammatory responses in the brain [20,21,22]. AT2 receptor activation counteracts most effects of the AT1 receptor by inhibiting cell proliferation and differentiation, promoting vasodilation, and reducing inflammation and oxidative stress [23]. DeNoble et al. reported that intracerebroventricular injection of renin frustrates the learning of a passive avoidance response in the rat. In their experiments, ACE inhibitor captopril and AT1 receptor antagonists EXP3312 and EXP3880 antagonized this effect in a dose-dependent manner. In their work, AT2 receptor blocker, PD123177, was not effective in preventing the renin-induced decrease in retention across a broad range of doses [24]. Conversely, Raghavendra et al. showed that both captopril and losartan improve memory in a retrieval active avoidance behavioral testing and only captopril improves learning. They attributed this effect of captopril to its interaction with the cholinergic system [3]. Based on this kind of observation, it was suggested that ACE inhibitors besides their antihypertensive effects might improve cognitive functions via brain neurotransmitter systems [25]. In this regard, Tota et al. suggested that the nootropic effect of ACE inhibitors is related to interference with the brain cholinergic system [26]. Moreover, Wilson et al. examined the role of the angiotensin 4 (Ang-IV) and its receptors and cholinergic systems in the nucleus basalis magnocellularis (Meynert in humans and primates) (NBM) of the rat. NBM is a main source of cholinergic innervation to cognitive areas of the brain including the hippocampus. They reported that the cholinergic and Ang-IV systems participate functionally in mediating cognitive processing via the NBM [12].

It is generally accepted that hippocampal acetylcholine is synthesized and released exclusively from the terminals of the long-axon afferents whose cell bodies reside in the basal forebrain [27]. More recently, Orta-Salazar et al. have shown the existence of ChAT and AChE as cholinergic neuron markers in the hippocampus of some animal species including rats [6]. To confirm the link between Ang-II and the cholinergic system, we evaluated ChAT expression in the hippocampus of hypertensive and normal rats. Our results indicated that renal hypertension in rats decreased the expression of ChAT in the hippocampus, an effect that was reversed by the treatment with captopril but not with losartan. ChAT is a transferase enzyme responsible for the synthesis of the neurotransmitter acetylcholine. Therefore, the reduction in the activity of this enzyme will reduce acetylcholine release in cholinergic synapses. This reduction can be one possible mechanism responsible for the memory impairment observed in renal hypertension. In earlier studies, researchers observed increased hippocampal acetylcholinesterase activity in Goldblatt hypertensive rats, which is in line with our findings [14]. Moreover, Cevikelli-Yakut et al used a rat Goldblott hypertention model to assess the improving effect of *Mirtus communis* extract on the cognitive function of the rats compared to ramipril in the MWM task. They reported that both *Mirtus communis* extract and ramipril improved the hypertensive rat’s spatial memory function. These researchers suggested that the inhibition of RAS in addition to its blood pressure-reducing effect may have a positive effect on the memory functions by decreasing the degradation of acetylcholine and increasing its activity [13]. In our experiments, we observed a significant increase in ChAT mRNA levels in the hippocampus of the captopril-treated hypertensive rats, but not in the losartan-treated group. It is possible that the intrinsic cholinergic neurons of the hippocampus play a role in spatial memory. Hence, captopril by increasing the ChAT mRNA levels can improve the spatial memory, while losartan that acts mainly via potentiating the neurogenesis in the dentate gyrus [28] has not shown any significant effect on the ChAT mRNA levels. In conclusion, the ability of ACE inhibitor captopril to facilitate cognitive processes in renovascular hypertensive rats might be related to ChAT overexpression in the hippocampus via AT1 receptors.
